# Neonatal Infection Due to SARS-CoV-2: An Epidemiological Study in Spain

**DOI:** 10.3389/fped.2020.580584

**Published:** 2020-10-23

**Authors:** Belén Fernández Colomer, Manuel Sánchez-Luna, Concepción de Alba Romero, Ana Alarcón, Ana Baña Souto, Fátima Camba Longueira, María Cernada, Zenaida Galve Pradell, María González López, M. Cruz López Herrera, Carmen Ribes Bautista, Laura Sánchez García, Elena Zamora Flores, Adelina Pellicer, Clara Alonso Díaz, Cristina Herraiz Perea, Dolores Sabina Romero Ramírez, Isabel de las Cuevas Terán, Isabel Pescador Chamorro, José Luis Fernández Trisac, Luis Arruza Gómez, Luis Miguel Cardo Fernández, Mª Jesús García García, Marta Nicolás López, Miryam Hortelano López, Mónica Riaza Gómez, Natalio Hernández González, Raquel González Sánchez, Sílvia Zambudio Sert, Susana Larrosa Capacés, Vanesa Matías del Pozo

**Affiliations:** ^1^Department of Neonatology, Central de Asturias University Hospital, Oviedo, Spain; ^2^Neonatology Department, Complutense University, Madrid, Spain; ^3^Division of Neonatology, Gregorio Marañón University Hospital, Madrid, Spain; ^4^Department of Neonatology, 12 de Octubre University Hospital, Madrid, Spain; ^5^Department of Neonatology, Sant Joan de Déu University Hospital, Barcelona, Spain; ^6^Department of Neonatology, Clinical Hospital de Santiago, Santiago de Compostela, Spain; ^7^Department of Neonatology, Vall d'Hebron University Hospital, Barcelona, Spain; ^8^Division of Neonatology, La Fe University and Polytechnic Hospital, Valencia, Spain; ^9^Department of Neonatology, Miguel Servet University Hospital, Zaragoza, Spain; ^10^Department of Neonatology, Regional de Málaga University Hospital, Málaga, Spain; ^11^Department of Neonatology, Cruces University Hospital, Baracaldo, Spain; ^12^Department of Neonatology, La Paz University Hospital, Madrid, Spain; ^13^Department of Neonatology, Virgen de la Salud University Hospital, Toledo, Spain; ^14^Department of Neonatology, Nuestra Señora de Candelaria University Hospital, Sta. Cruz de Tenerife, Spain; ^15^Department of Neonatology, Marqués de Valdecilla University Hospital, Santander, Spain; ^16^Department of Neonatology, A Coruña University Hospital Complex, Coruña, Spain; ^17^Department of Neonatology, Clinical Hospital San Carlos, Madrid, Spain; ^18^Department of Neonatology, Denia Hospital, Denia, Spain; ^19^Neonatology Unit, San Pedro de Alcántara Hospital, Cáceres, Spain; ^20^Department of Neonatology, Germans Trias i Pujol University Hospital, Badalona, Spain; ^21^Department of Neonatology, Segovia Assistance Complex, Segovia, Spain; ^22^Department of Neonatology, Montepríncipe Hospital, Madrid, Spain; ^23^Department of Pediatrics, Zamora Assistance Complex, Zamora, Spain; ^24^Department of Neonatology, Quironsalud Hospital, Madrid, Spain; ^25^Department of Neonatology, Igualada Hospital, Barcelona, Spain; ^26^Department of Neonatology, Sant Joan de Reus Hospital, Tarragona, Spain; ^27^Department of Neonatology, University Clinic Hospital, Valladolid, Spain

**Keywords:** coronavirus disease 2019, SARS-CoV-2 infection, neonates, epidemiology–descriptive, hospital-acquired infection, community-acquired infection

## Abstract

**Objective:** Coronavirus disease 2019 (COVID-19) cases caused by the severe acute respiratory syndrome coronavirus 2 (SARS-CoV-2) continue to increase worldwide. Although some data from pediatric series are available, more evidence is required, especially in neonates, a group with specific characteristics that deserve special attention. This study aimed to describe general and clinical characteristics, management, and treatment of postnatal-acquired (community and nosocomial/hospital-acquired) COVID-19 neonatal cases in Spain.

**Methods:** This was a national prospective epidemiological study that included cases from a National Registry supported by the Spanish Society of Neonatology. Neonates with postnatal SARS-CoV-2 infection were included in this study. General data and infection-related information (mode and source of transmission, age at diagnosis, clinical manifestations, need for hospitalization, admission unit, treatment administered, and complementary studies performed, hospital stay associated with the infection) were collected.

**Results:** A total of 40 cases, 26 community-acquired and 14 nosocomial were registered. Ten were preterm newborns (2 community-acquired and 8 nosocomial COVID-19 cases). Mothers (in both groups) and healthcare workers (in nosocomial cases) were the main source of infection. Hospital admission was required in 22 community-acquired cases [18 admitted to the neonatal intermediate care unit (NIMCU) and 4 to the neonatal intensive care unit (NICU)]. Among nosocomial COVID-19 cases (*n* = 14), previously admitted for other reasons, 4 were admitted to the NIMCU and 10 to the NICU. Ten asymptomatic patients were registered (5 in each group). In the remaining cases, clinical manifestations were generally mild in both groups, including upper respiratory airways infection, febrile syndrome or acute gastroenteritis with good overall health. In both groups, most severe cases occurred in preterm neonates or neonates with concomitant pathologies. Most of the cases did not require respiratory support. Hydroxychloroquine was administered to 4 patients in the community-acquired group and to 2 patients in the nosocomial group. Follow-up after hospital discharge was performed in most patients.

**Conclusions:** This is the largest series of COVID-19 neonatal cases in Spain published to date. Although clinical manifestations were generally mild, prevention, treatment, and management in this group are essential.

## Introduction

The first cases of pneumonia caused by the severe acute respiratory syndrome coronavirus 2 (SARS-CoV-2) were detected in December 2019 in Hubei Province, China, and spread rapidly throughout the country and then the world (1). As of August 26, 2020, there had been over 24 million confirmed cases, and more than 800,000 deaths due to the disease (2). The risk of death due to coronavirus disease 2019 (COVID-19) increases exponentially with age. Thus, mortality rates are in the order of 0.12% in children, but as high as 14.8% in older individuals (3, 4). The reason why children are less susceptible to COVID-19 than adults remains unclear (5), but new concerns about a novel severe Kawasaki-like disease related to COVID-19 have emerged (6), demonstrating that the infection and its putative long-term consequences in this population, including neonates, should be carefully studied.

Although COVID-19 infections among pregnant women (7, 8) and newborns (9) have been reported, vertical intrauterine transmission is still a controversial issue (10, 11). In certain cases, such as the one described by Kirtsman et al. (12), SARS-CoV-2 RNA was detected from a nasopharyngeal swab sample collected on the day of birth before skin-to-skin contact with the mother. However, since positivity could not be confirmed in the cord tissue, the authors determined this case to be a probable case of congenital SARS-CoV-2 infection (as opposed to a confirmed case). Other new published cases have been classified as transplacental transmission, since they show placental involvement and neonatal infection by detection of the virus by PCR in a nasopharyngeal swab at birth (13, 14) and even neonatal viremia (15). Shah et al. have established a classification system for maternal-fetal-neonatal SARS-CoV-2 infections, defining the criteria that infections should meet to be classified as congenital, intrapartum, or postpartum (16).

Other data, however, suggest possible horizontal and nosocomial transmission modes for the virus in neonates. Recently, several cases of SARS-CoV-2 isolated in breastmilk collected from infected nursing mothers have been reported (17, 18). In one of the cases, SARS-CoV-2 RNA was detected in milk at days 10, 12, and 13, coinciding with SARS-CoV-2 positive diagnostic testing of the newborn, who developed mild COVID-19 symptoms from day 10 on. However, as described by Chambers et al. detection of viral RNA by reverse transcriptase–polymerase chain reaction (RT-PCR) does not equate with infectivity (19). Overall, these data highlight the importance of prevention and early detection and follow-up in neonates, in whom management and treatment still need to be defined.

Like in other age groups, major transmission routes in neonates are respiratory droplets, contact transmission and aerosol transmission (20). Data in this population are scarce, from small series included in larger studies on pediatric cases, in which, unlike adults, clinical manifestations have been reported to be mild (21). Interestingly, the proportion of severe and critical cases reported in children is inversely correlated with their age, and infections in infants <1 year old have been more severe (22). On the other hand, neonates constitute an heterogeneous group in terms of age, with specific clinical and epidemiological features. In this regard, preterm newborns are especially vulnerable for viral infections, as demonstrated with other viruses such as respiratory syncytial virus (RSV), influenza or cytomegalovirus, and the clinical manifestations they develop are not always mild (23–26).

Since the detection of the first positive case in neonates in Spain (27), the Spanish Society of Neonatology aimed to expand the knowledge of COVID-19 in this population and, in order to improve the well-being and care of their patients during SARS-CoV-2 pandemic, sponsored the creation of a National Registry. This study describes a large series of neonatal patients from the National Registry with confirmed or probable postpartum SARS-CoV-2 infection (including both nosocomial and community-acquired cases) according to the Shah et al. classification system (16).

## Materials and Methods

The Spanish Society of Neonatology (*Sociedad Española de Neonatolog*í*a*, SENEO) developed a nationwide prospective online case registration system aimed at collecting real-time data of neonates born to mothers diagnosed with COVID-19 during pregnancy or in the immediate postpartum period and neonates with postnatal (community-acquired and nosocomial) disease. This study focused on the latter cases. SENEO executive committee members reviewed the literature and designed the items that should be entered into the database, which was accessible from the SENEO website to all neonatology departments in Spain. Staff neonatologists from each department were responsible for entering data on different variables of the clinical characteristics of mothers and neonates.

Specific precautions taken during delivery and the postpartum period were not recorded, but all procedures were performed according to the guidelines and other recommendations summarized in an official technical document entitled “Management of pregnant women and newborns with COVID-19” issued by the Spanish Ministry of Health in March (last version June 17, 2020) (28).

Diagnosis and management, visitor protocol, and isolation measures of newborns hospitalized with COVID-19 were applied in accordance with clinical guidelines of the SENEO (29).

The RT-PCR tests most frequently used in the laboratories of the largest participating hospitals were the Allplex™ 2019nCoV Assay (Seegene), TaqMan™ 2019-nCoV Assay Kit v1 (ThermoFisher Scientific), TaqPath™ COVID-19 Combo Kit (Applied Biosystems), and SARS-COV-2 Realtime PCR kit (Vircell).

The study was approved by the Clinical Research Ethics Committee of the Principality of Asturias. Written informed consent was obtained from the neonates' parents.

Strengthening the Reporting of Observational Studies in Epidemiology (STROBE) recommendations were followed for this study (30).

### Data Collection

An online form consisting of 75 variables was created. In this form, general information was collected on perinatal data and type of feeding, infection-related information [mode (hospital-acquired/nosocomial or community-acquired) and source of transmission (mother, other relative, healthcare or unknown), age at diagnosis, clinical manifestations, hospital admission requirement, admission unit, treatment applied, complementary studies performed, and hospital stay associated with the infection]. Whether follow-up upon hospital discharge was performed, and an empty space for additional participant's commentaries were also included.

A total of 79 hospitals participated in the study, 21 of which (six from the Community of Madrid and five from the Community of Catalonia) registered SARS-CoV-2 positive neonatal cases (Supplementary Table 1).

### Statistical Analysis

Quantitative data are presented as means and standard deviation or medians and interquartile ranges (IQR). Frequencies and percentage were used to estimate the qualitative data. Student's *t*-test or the Kruskal–Wallis test were used for the comparison of quantitative variables, and the Chi-square test or Fisher's exact test were used for the comparison of qualitative variables. Statistical analyses were performed using SPSS Statistics (version 23.0, IBM Corp., Armonk, NY, USA).

## Results

From the opening of the National Registry (3 April 2020) to the cut-off date (18 May 2020), 40 cases were registered in 21 hospitals in Spain; 26 community-acquired COVID-19 cases and 14 nosocomial cases.

Among the total 26 community-acquired cases, 24 were full-term neonates and 2 were preterm (Table 1). Most of them were male (*n* = 15, 57.5%) and presented normal neonatal characteristics. Maternal lactation (alone or combined) rate was 96.15%. In the group of nosocomial cases, 6 were full-term neonates and 8 were preterm infants (3 very preterm infants) (Table 1). As in the community-acquired COVID-19 group, most of them were male (*n* = 10, 71.4%) and maternal lactation (alone or combined) was the most frequent type of feeding (*n* = 11, 78.57%). When both groups were compared, the neonates in the nosocomial-acquired COVID-19 group had, as expected, a lower gestational age, and lower anthropometric values. A lower incidence of delayed cord clamping and less breastfeeding were also recorded, although maternal lactation rates were similar (Table 1).

**Table 1:** General characteristics of the study population.

**Variable**	**Community-acquired COVID-19 cases *n* = 26**	**Nosocomial COVID-19 cases *n* = 14**	***p*-value**
**Anthropometrics, mean ± SD**			
Weight (gr)	3131 ± 623	2626 ± 744	0.001
Length (cm)	49.7 ± 3.3	45.7 ± 3.6	0.002
Cranial perimeter (cm)	34 ± 2.0	31.8 ± 2.5	0.01
Male, *n* (%)	15 (57.6)	10 (71.4)	NS
Gestational age (weeks), mean ± SD	39 ± 2.2	35.1 ± 3.9	0.001
**Prematurity**, ***n*** **(%)**			
<37 weeks	2 (7.7)	8 (57.1)	0.001
≤32 weeks	2 (7.7)	3 (21.5)	NS
**Apgar score, median (IQR)**			
1 min	9 (8-9)	7 (5-9)	NS
5 min	10 (9-10)	9 (7.7-10)	NS
Immediate skin-to-skin contact, *n* (%)	21 (80.8)	9 (64.3)	NS
Delayed cord clamping, *n* (%)	18 (69.2)	6 (42.9)	0.07
**Feeding**, ***n*** **(%)**			NS
Maternal lactation	14 (53.8)	7 (50.0)	
Mixed feeding	11 (42.3)	4 (28.6)	
Formula-feeding	1 (3.8)	3 (21.4)	
**Type of feeding**, ***n*** **(%)**			0.01
Breastfeeding	16 (61.5)	3 (21.4)	
Bottle feeding	2 (7.7)	3 (21.4)	
Both	8 (30.8)	3 (21.4)	
Nasogastric tube	–	5 (35.7)	

*NS, not significant*.

### Community-Acquired COVID-19 Cases

Infection-related characteristics/variables of the community-acquired COVID-19 cases are shown in Table 2.

**Table 2:** Infection-related characteristics in community-acquired COVID-19 cases.

**Variable**	**Community-acquired COVID-19 cases *n* = 26**
Age at diagnosis (days), median (IQR)	17 (11–26)
Hospital admission, *n* (%)	22 (84.6)
Neonatal intensive care unit	4 (15.4)
Neonatal intermediate care unit	18 (69.2)
**Potential source of infection**, ***n*** **(%)**	
Mother	16 (61.5)
Other relative	8 (30.8)
Unknown	2 (7.7)
**Clinical signs and symptoms**, ***n*** **(%)**	
Asymptomatic cases	5 (19.2)
Fever	13 (50.0)
Cough	6 (23.1)
Phlegm	5 (19.2)
Hypoxemia	5 (19.2)
Tachypnea	4 (15.4)
Apathy	3 (11.5)
Vomiting	3 (11.5)
Apnea	1 (3.8)
Diarrhea	1 (3.8)
Exanthema	1 (3.8)
**Neonatal diagnostic test**, ***n*** **(%)**	
PCR (nasopharyngeal exudate/aspirate)	26 (100)
**Laboratory test**, ***n*** **(%)**	
Hemoglobin (g/dL), median (IQR)	13.5 (12–16.8)
Leucopenia (<5,000/mm^3^), *n* (%)	3/21 (14.3)
Lymphopenia (<3,000/mm^3^), *n* (%)	4/21 (19.0)
Thrombopenia (<100,000/mm^3^), *n* (%)	0/21
C-reactive protein (mg/dL), median (IQR)	0.5 (0.1–1.7)
Procalcitonin (ng/mL), median (IQR)	0.17 (0.11–0.28)
AST (U/L), median (IQR)	46 (28–74)
ALT (U/L), median (IQR)	23 (17–28)
Chest X-ray, *n* (%)	12 (46.1)
Normal	7 (58.3)
Ground-glass opacity	2 (16.67)
Interstitial abnormalities	1 (8.33)
Perihilar thickening	1 (8.33)
Atelectasis	1 (8.33)
**Respiratory support**, ***n*** **(%)**	
None	21 (81.7)
Oxygen	5 (19.2)
High-flow nasal cannula	2 (7.7)
Nasal CPAP	1 (3.8)
Low-flow nasal cannula	1 (3.8)
**Pharmacotherapy**, ***n*** **(%)**	
None	19 (73.1)
Antibiotics	3 (11.5)
Hydroxychloroquine	1 (3.8)
Antibiotics + hydroxychloroquine	2 (7.7)
Azithromycin + hydroxychloroquine + lopinavir/ritonavir	1 (3.8)
**Length of hospital stay, median (IQR)**	4 (2-6.2)
**Follow-up upon hospital discharge**, ***n*** **(%)**	22 (84.6)

*ALT, alanine aminotransferase; AST, aspartate aminotransferase; COVID-19, coronavirus disease 2019; CPAP, continuous positive airway pressure; PCR, polymerase chain reaction*.

In this group, median age at diagnosis was 17 (11.5–26.5) days. All cases were diagnosed by polymerase chain reaction (PCR) for SARS-CoV-2 in nasopharyngeal exudates (one case was also evaluated in a urine sample that resulted negative). Of the 26 cases, 5 remained asymptomatic and were detected by the study of close contacts (*n* = 2, 40%), by hospital admission due to causes other than COVID-19, and mandatory PCR test for SARS-CoV-2 in all admissions according to hospital admission regulations (*n* = 2, 40%; hospital admission due to jaundice), or by follow-up after being negative at birth and with a SARS-CoV-2 positive mother (*n* = 1, 20%) (follow-up performed from birth due the mother being SARS-CoV-2-positive).

**Figure 1:**
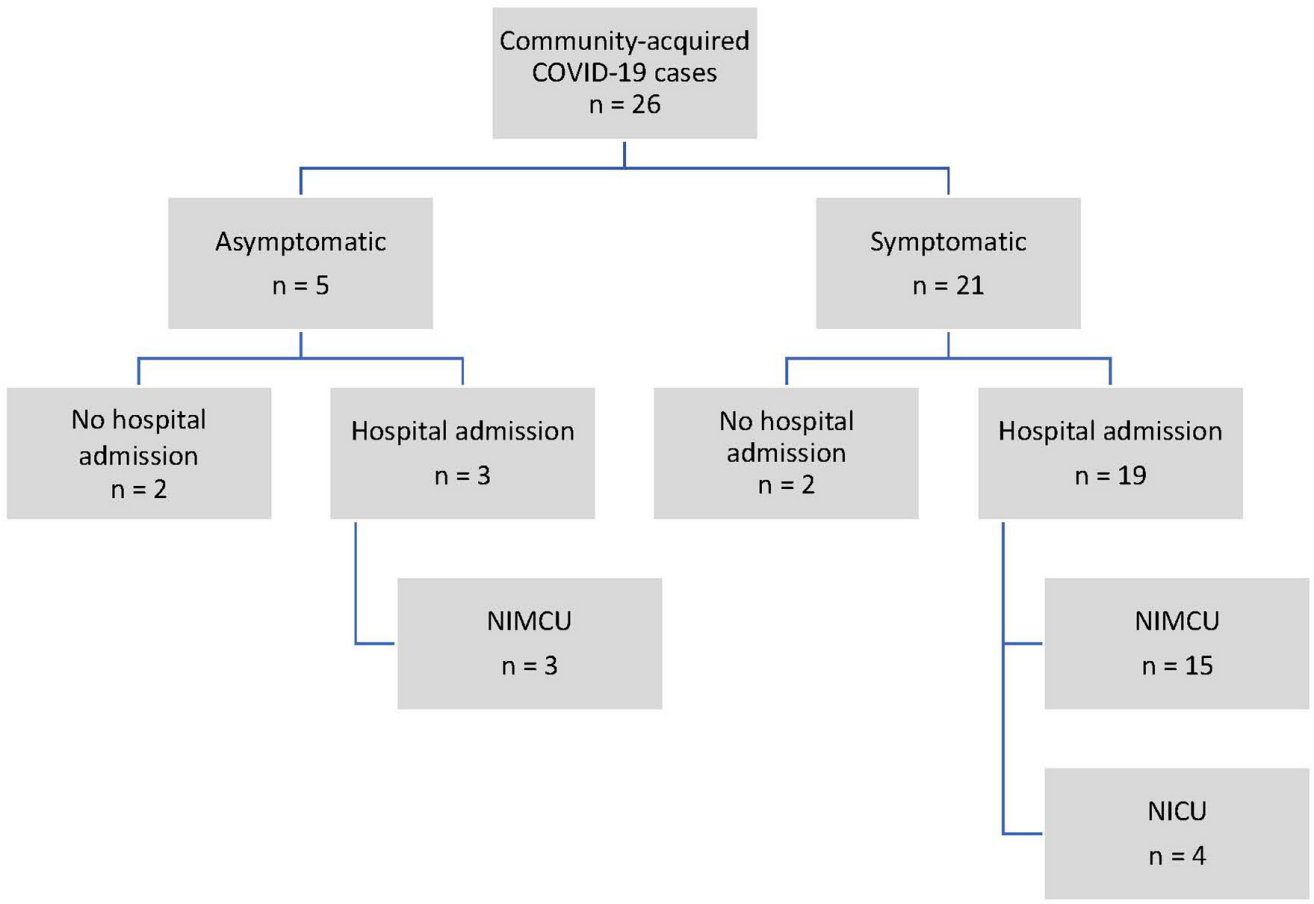
Community-acquired COVID-19 case distribution. COVID-19, coronavirus disease 2019; NICU, neonatal intensive care unit; NIMCU, neonatal intermediate care unit.

A total of 22 neonates were admitted to the hospital, 19 for COVID-19-related symptoms and 3 asymptomatic cases (2 admitted due to jaundice and 1 for a 24-h follow-up) (Figure 1). Eighteen patients were admitted to the neonatal intermediate care unit (NIMCU) and 4 to the neonatal intensive care unit (NICU). Of the latter, 2 were preterm newborns (32-week-old twins) with pneumonia and 2 were full-term newborns with bronchiolitis (co-infection with rhinovirus and suspected primary ciliary dyskinesia) and apnea, respectively. Among those who did not require hospital admission, 2 presented fever with good overall health and 2 remained asymptomatic. In all cases, follow-up visits (by telephone or in person) were carried out. Mothers (*n* = 16, 61.5%), followed by other relatives involved in the neonate's care, were the main possible source of infection.

Overall, clinical manifestations were mild, and clinical spectrum included upper respiratory airways infections (*n* = 11), febrile syndrome (*n* = 4), acute gastroenteritis (*n* = 1), apnea (*n* = 1), and mild respiratory distress (*n* = 1). The most severe manifestations occurred in the 2 preterm neonates with pneumonia and in the neonate with bronchiolitis due to rhinovirus co-infection.

Laboratory tests were performed in 21 neonates. Three cases of leukopenia and four cases of lymphopenia were detected (concurrently in two cases). No other laboratory test abnormalities were detected. Chest X-rays were performed only in 12 patients, 7 of which were normal. In the 5 remaining cases, SARS-CoV-2 infection-related radiological patterns were identified (atelectasis, a non-typical SARS-CoV-2 infection-related radiological pattern, was observed in the neonate with bronchiolitis). Abdomen X-ray and echocardiography were performed in 2 cases, respectively, both of which were normal.

Respiratory support was required in few cases and in those who did need it, oxygen and non-invasive systems [high and low-flow nasal cannula and nasal continuous positive airway pressure (CPAP)] were used briefly.

Regarding treatment, oral hydroxychloroquine was administered on a compassionate use basis to 4 patients (patients with bronchiolitis, with pneumonia and with acute respiratory distress syndrome). Three of these patients presented moderate/severe conditions and had concomitant pathologies. In all of them, the disease evolution was favorable.

Finally, control PCR was carried out in 8 cases with a median of 21 days after diagnosis. Negative results were obtained in all cases. In the remaining cases (*n* = 18), no PCR control was performed since patients were asymptomatic or very mildly clinical, and in some cases because diagnostic tests were not available in the early stages of the pandemic. In 22 neonates, clinical follow-up was performed, or it is still ongoing. No additional issues have been reported to date.

### Nosocomial COVID-19 Cases

The 14 cases occurred in 9 hospitals, as follows: single cases in 7 centers, 4 cases in 1 hospital (2 involving twins and another 2 isolated cases), and 3 cases in another hospital (1 isolated case and 2 cases in the same outbreak).

Infection-related characteristics/variables of the nosocomial COVID-19 cases are shown in Table 3.

**Table 3:** Infection-related characteristics in nosocomial (hospital-acquired) COVID-19 cases.

**Variable**	**Nosocomial COVID-19 cases *n* = 14**
Age at diagnosis (days), median (IQR)	14.5 (7–43)
**Hospital unit**, ***n*** **(%)**	
Neonatal intensive care unit	4 (28.6)
Neonatal intermediate care unit	10 (71.4)
**Potential source of infection**, ***n*** **(%)**	
Mother	6 (42.9)
Healthcare workers	6 (42.9)
Unknown	2 (14.3)
**Clinical signs and symptoms**, ***n*** **(%)**	
Asymptomatic cases	5 (35.7)
Fever	5 (35.7)
Cough	3 (21.4)
Hypoxemia	2 (14.3)
Tachypnea	3 (21.4)
Apathy	1 (7.1)
Vomiting	1 (7.1)
Diarrhea	1 (7.1)
**Neonatal diagnostic test**, ***n*** (%)^*^	
PCR (nasopharyngeal exudate/aspirate)	13 (92.9)
PCR (bronchoalveolar lavage)	1 (7.1)
**Laboratory test**, ***n*** **(%)**	
Hemoglobin (g/dL), median (IQR)	12.6 (10.6–17.2)
Leucopenia (<5,000/mm^3^), *n* (%)	1/12 (8.3)
Lymphopenia (<3,000/mm^3^), *n* (%)	2/12 (16.7)
Thrombopenia (<100,000/mm^3^), *n* (%)	1/12 (8.3)
C reactive protein (mg/dL), median (IQR)	1.2 (0.4–3.9)
Procalcitonin (ng/mL), median (IQR)	0.26 (0.15–2.1)
AST (U/L), median (IQR)	41 (21.7–63.5)
ALT (U/L), median (IQR)	24 (15.7–47)
Chest X-ray, *n* (%)	8 (57.1)
Normal	4 (50.0)
Ground-glass opacity	1 (12.5)
Interstitial abnormalities	2 (25.0)
Perihiliar thickening	1 (12.5)
**Respiratory support**, ***n*** **(%)**	
None	12 (85.7)
Oxygen	2 (14.2)
High-flow nasal cannula	1 (7.1)
Conventional intermittent positive pressure ventilation	1 (7.1)
**Pharmacotherapy**, ***n*** **(%)**	
None	10 (71.4)
Antibiotics	2 (14.2)
Hydroxychloroquine	2 (14.2)
**Hospital stay length, median (IQR)**	–
**Follow-up upon hospital discharge**, ***n*** **(%)**	4 (28.6)

* *All PCR tests performed in feces or urine resulted negative*.

*ALT, alanine aminotransferase; AST, aspartate aminotransferase; COVID-19, coronavirus disease 2019; CPAP, continuous positive airway pressure; PCR, polymerase chain reaction*.

In this group, median age at diagnosis was 14.5 (7.2–43) days. Cases were diagnosed by polymerase chain reaction (PCR) for SARS-CoV-2 in nasopharyngeal exudates (*n* = 13, 92.9%) or bronchoalveolar lavage (*n* = 1, 7.1%; intubated). In 3 patients, PCRs for SARS-CoV-2 were also performed in feces samples and in urine in one case, all of which were negative. Of the 14 cases, 5 remained asymptomatic and were detected by the study of close contacts.

Pre-existing hospital admission was due to prematurity in 8 cases, and among those born at term (*n* = 6; 42.85%), hospital admission was due to surgical-related causes in 2 patients, hypoxic-ischemic encephalopathy in 1 patient, Gram-negative sepsis in 1 patient, dehydration in 1 patient, and for social reasons in 1 patient. Ten newborns had been admitted to the NIMCU and 4 to the NICU.

In symptomatic neonates (*n* = 9; 64.29%), main clinical diagnoses were upper respiratory airway infection in 5 patients, febrile syndrome in 2 patients, acute gastroenteritis in 1 patient, and dehydration in 1 patient.

Mothers (*n* = 6, 42.9%) and health professionals (*n* = 6, 42.9%) were the main possible source of infection.

Laboratory tests were performed in 12 neonates. One case of leukopenia, 2 cases of lymphopenia and 1 case of thrombopenia (co-infection with a Gram-negative bacillus) were detected. No other laboratory test abnormalities were detected. Chest X-rays were performed only in 8 patients, half of which were normal. In the four remaining cases, SARS-CoV-2 infection-related radiological patterns were identified.

COVID-19-related non-invasive respiratory support was required in only one case. Conventional intermittent positive pressure ventilation was applied previously to SARS-CoV-2 infection in one case.

Regarding treatment, oral hydroxychloroquine was administered to 2 patients (preterm twins with severe combined immunodeficiency and congenital cytomegalovirus infection).

Finally, a control PCR was carried out in 10 cases, between 2 and 30 days (median 11 days) after diagnosis. Negative results were obtained in all cases. Clinical follow-up was performed or is still ongoing in all 14 neonates with nosocomial disease. No additional issues have been reported to date.

## Discussion

In this study, a large series of neonatal cases with community-acquired or nosocomial (hospital-acquired) COVID-19 is presented.

Firstly, and similar to reports from other series including neonates (31), a prevalence of male patients has been determined, although the implication of this finding remains unclear. To date, although it has been suggested among adults that men with COVID-19 are at higher risk for worse outcomes and death regardless of their age, no differences in terms of prevalence have been registered (32).

In Spain, the first neonatal case was detected in March 2020, possibly due to horizontal transmission between mother and newborn, who were both admitted to hospital (27). Major transmission routes in neonates, as in other age groups, are respiratory droplets, contact transmission and aerosol transmission (20). As expected, in community-acquired COVID-19 cases, the mother or individuals involved in the neonate's care were the major source of infection. In nosocomial cases, considering that most of the Spanish neonatal units operate an open-door visiting policy and provide developmental care, possible transmission between parents and newborns, especially during the asymptomatic phase of infection (33), constitutes one of the main potential risks. Moreover, healthcare professionals involved in the neonate's care are also a key element in nosocomial transmission, especially in times of high prevalence, underlining the importance of adequate personal protective equipment and virological tests to assess their infection status (34, 35). In our series, healthcare workers were the source of infection in 42.9% of the nosocomial cases. In a review by Lavizzari et al. on the management of children of COVID-19 positive mothers performed in 20 countries across different continents, the authors concluded that the lack of evidence at the beginning of the pandemic led to good communication in the exchange of information between countries. However, they emphasized that future guidance should be built on high-level evidence, rather than expert consensus (36).

COVID-19 is usually milder in children than in adults, and especially in neonates, and may be accompanied by non-specific symptoms (20). In our series, most of the cases were admitted to the NIMCU with a median length of hospital stay of 4 days and followed this pattern of milder infection, in line with previous studies (9, 37). The rate of hospitalization in the neonatal unit was high, due to the unknown course of the disease at that time. Nevertheless, in the light of current knowledge, in mild forms, joint mother-child hospitalization in single rooms in the maternity ward may be evaluated, in order to avoid overuse of the neonatal hospitalization units (NIMCU, NICU) by restricting admission to moderate and severe cases. However, certain neonatal patients, such as preterm newborns or neonates with concomitant chronic diseases, may be specially affected by the disease and may develop more severe clinical manifestations, as observed here and also by other authors (38). In our study, patients with concomitant chronic diseases presented progressive respiratory distress and hypoxemia, and required admission to the NICU, along with respiratory support and/or oxygen therapy. Indeed, both pneumonia cases occurred in 32-week-old preterm newborns. Viral co-infection is another scenario that must be especially considered in this population, specifically at times of the year in which respiratory viruses (RSV, rhinovirus, influenza virus, etc.) are highly prevalent (autumn and winter) (39). Here, we reported one co-infection with rhinovirus that required intensive care, oxygen therapy, and ventilatory support, showing that more severe manifestations may occur in subjects with concomitant viral infections.

As in the adult population, asymptomatic cases can be also found among neonates, constituting a set of cases who can silently spread the virus and can be easily neglected in epidemic prevention (40). This becomes especially relevant in cases of admission to neonatal care units for causes other than COVID-19 (surgery, jaundice etc.). In this context, and in times of virus circulation, virological assessment prior to admission, especially in open neonatal units (the most common type in Spain), is highly recommended.

The most common diagnostic tool in our study was PCR testing in nasopharyngeal exudates; all PCR tests performed in feces and/or urine samples were negative. Despite these results, we believe that fecal analysis might be relevant for determining virus transmission, particularly in the hospital environment. Indeed, a recent report identified cases of COVID-19 patients in whom, despite negative nasopharyngeal follow-up PCR tests, continued to have SARS-CoV-2 detected in stool for weeks afterwards (41).

In line with previous studies (42), laboratory test results in our patients showed no relevant abnormalities: only 7 cases presented white cell disturbances, while liver function markers and acute phase reactants results (C-reactive protein and procalcitonin) were within normal levels. Nevertheless, a consistent pattern of laboratory abnormalities has yet to be observed in children with confirmed COVID-19. Radiological patterns observed here are in line with those described in other pediatric series (10, 31).

Regarding treatment, hydroxychloroquine was administered on a compassionate use basis in 6 cases, due to its potential antiviral effect suggested in adults (43). All these patients were severe cases diagnosed at critical moments during the pandemic in Spain (March/April 2020): 2 pneumonias, 1 case of acute respiratory distress syndrome and 3 cases with concomitant conditions (one case of bronchiolitis with suspected primary ciliary dyskinesia who also received azithromycin and lopinavir/ritonavir, and two cases of severe combined immunodeficiency). The lack of studies in the pediatric population should limit hydroxychloroquine use to clinical trials.

Finally, following the recommendations issued by the Spanish Neonatal Society (29), clinical follow-up upon hospital discharge at least within the first 14 days was performed in all cases. No data are available for those who were still hospitalized on the date of study cut-off. We believe that follow-up is an essential part of the management of these patients, and will help to improve our mid- and long-term understanding of the disease.

This study has also some limitations. Firstly, urine and feces samples could not be collected in all cases, and consequently, PCR diagnosis in those specimens was not available for all neonates. Secondly, radiological images were missing in the database. Thirdly, no data were collected on other problems during pregnancy or in the newborn. This decision was taken to avoid overloading researchers and to reduce data loss and bias. Finally, follow-up data were incomplete, since at the study cut-off date (28^th^ May 2020), several patients had not yet been discharged. Despite these limitations, our study provides important results in a large sample population.

In conclusion, the first large series of COVID-19 neonatal cases registered in Spain included in this study provides a general and relevant picture of the pandemic situation in this specific population.

## Data Availability Statement

All datasets generated for this study are included in the article/Supplementary Material.

## Ethics Statement

The studies involving human participants were reviewed and approved by the Ethics Committee of Regional Clinical Research of the Principality of Asturias. Written informed consent to participate in this study was provided by the participants' legal guardian/next of kin.

## Author Contributions

BF and MS-L were responsible for the study design, data acquisition, analysis, and interpretation. CA contributed to data acquisition and analysis, article drafting, and revision. All authors reviewed and approved the manuscript.

## Conflict of Interest

The authors declare that the research was conducted in the absence of any commercial or financial relationships that could be construed as a potential conflict of interest.

## Consortium

Manuel Sánchez Luna^1^, Isabel Izquierdo Macián^2^, Ester Sanz López^1^, Segundo Rite Gracia^3^, Martín Iriondo Sanz^4^, Alejandro Pérez Muñuzuri^5^, Tomás Sánchez Tamayo^6^, Belén Fernández Colomer^7^, José Luis Leante Castellanos^8^ and Ana Remesal Escalero^9^

^1^Hospital General Universitario Gregorio Marañón de Madrid, Madrid, Spain
^2^Hospital Universitari i Politècnic La Fe de Valencia, Valencia, Spain
^3^Hospital Materno Infantil Miguel Servet de Zaragoza, Zaragoza, Spain
^4^Hospital Univeristari Sant Joan de Déu de Barcelona, Barcelona, Spain
^5^Hospital Clínico Universitario de Santiago de Compostela, Santiago, Spain
^6^Hospital Regional Universitario de Málaga, Málaga, Spain
^7^Hospital Universitario Central de Asturias, Oviedo, Spain
^8^Hospital Universitario Santa Lucía de Cartagena, Cartagena, Spain
^9^Hospital Universitario de Salamanca, Salamanca, Spain.
