# Supplementary material for: Neonatal Infection Due to SARS-CoV-2: An Epidemiological Study in Spain
Source: Front Pediatr. 2020 Oct 23;8:580584. doi: 10.3389/fped.2020.580584 (PMC7644848; doi:10.3389/fped.2020.580584)
Supplement: Supplementary file 1 [file Table_1.docx]

**Supplementary Table S1**. List of participating hospitals and their contribution in number of cases.

| **Participant hospitals** | **Number of cases registered**  ***n* (%)** |
| --- | --- |
| La Paz University Hospital (Madrid) | 9 (22.5) |
| Virgen de la Salud University Hospital (Toledo) | 4 (10.0) |
| Germans Trias i Pujol University Hospital (Badalona, Barcelona) | 3 (7.5) |
| Quironsalud Hospital (Madrid) | 3 (7.5) |
| Segovia Assistance Complex (Segovia) | 3 (7.5) |
| Gregorio Marañón University Hospital (Madrid) | 2 (5.0) |
| Sant Joan de Deu University Hospital (Esplugues de Llobregat, Barcelona) | 2 (5.0) |
| 12 de Octubre University Hospital (Madrid) | 1 (2.5) |
| Clinical Hospital San Carlos (Madrid) | 1 (2.5) |
| Montepríncipe Hospital (Madrid) | 1 (2.5) |
| Igualada Hospital (Barcelona) | 1 (2.5) |
| Vall d'Hebron University Hospital (Barcelona) | 1 (2.5) |
| Sant Joan de Reus Hospital (Reus, Tarragona) | 1 (2.5) |
| A Coruña University Hospital (A Coruña) | 1 (2.5) |
| San Pedro de Alcántara Hospital (Cáceres) | 1 (2.5) |
| Nuestra Señora de Candelaria University Hospital (Santa Cruz de Tenerife) | 1 (2.5) |
| Marqués de Valdecilla University Hospital (Santander) | 1 (2.5) |
| Denia Hospital (Valencia) | 1 (2.5) |
| La Fe University and Polytechnic Hospital (Valencia) | 1 (2.5) |
| Clinical University Hospital de Valladolid (Valladolid) | 1 (2.5) |
| Zamora Assistance Complex (Zamora) | 1 (2.5) |
| **TOTAL** | **40 (100)** |
